# IFN-τ mediated miR-26a targeting PTEN to activate PI3K/AKT signalling to alleviate the inflammatory damage of bEECs

**DOI:** 10.1038/s41598-022-12681-9

**Published:** 2022-06-07

**Authors:** Junfeng Liu, Qin Liang, Tianyang Wang, Bei Ma, Xin Wang, Ping Li, Aftab Shaukat, Xuefeng Guo, Ganzhen Deng

**Affiliations:** 1 College of Animal Science and Technology, Tarim University, Alar, Xinjiang 843300 People’s Republic of China; 2 Engineering Laboratory of Tarim Animal Diseases Diagnosis and Control, Xinjiang Production & Construction Corps, Tarim University, Alar, Xinjiang 843300 People’s Republic of China; 3 Department of Clinical Veterinary Medicine, College of Veterinary Medicine, Huazhong Agricultural University, Wuhan, 430070 People’s Republic of China; 4 Key Laboratory of Agricultural Animal Genetics, Breeding and Reproduction, Education Ministry of China, Huazhong Agricultural University, Wuhan, 430070 People’s Republic of China

**Keywords:** Cell biology, Drug discovery, Immunology, Diseases

## Abstract

Endometritis is the failure of embryo implantation and an important cause of infertility in dairy cows. IFN-τ is a type I interferon unique to ruminants. In regulating the process of inflammatory response, IFN-τ can be expressed through MicroRNAs (miRNAs) to regulate the process of inflammation. However, IFN-τ regulates lipopolysaccharide (LPS)-induced inflammatory injury of bEECs through the highly conserved miR-26a in mammals, and the mechanism remains unclear. Bovine endometrial epithelial cells (bEECs)were isolated and cultured to establish an inflammatory injury model. RT–qPCR and ELISA were used to detect the secretion of inflammatory factors. Dual-luciferase assays and target gene silencing assays determine the regulatory role of miRNAs. The target protein was detected by immunofluorescence and western blotting. This study showed that the expression of miR-26a was significantly down-regulated in mouse endometrium inflammatory injury tissue and LPS stimulated bEECs; and IFN-τ reversed the expression of miR-26a. The study also showed that the overexpression of miR-26a significantly inhibited the secretion of pro-inflammatory cytokines IL-1β, IL-6 and TNF-α. In addition, studies have shown that miR-26a inhibits its translation by targeting PTEN 3′-UTR, which in turn activates the Phosphatidylinositide 3-kinases/protein kinase B (PI3K/AKT) pathway, so that nuclear factor kappa-B (NF-κB) signaling is inhibited. In summary, the results of this study further confirm that IFN-τ as an anti-inflammatory agent can up-regulate the expression of miR-26a and target the PTEN gene to inhibit the inflammatory damage of bEECs.

## Introduction

The endometrium can effectively prevent the invasion of pathogenic bacteria and is the first barrier against infection by pathogenic microorganisms^1^. Endometritis is one of the main causes of endometrial damage, and it is a common disease in reproductive system infections and one of the main causes of miscarriage in dairy cows^2,3^. The molecular mechanisms of endometritis and the endometrial injury pathways remain elusive. Despite decades of research, the incidence of endometritis remains high (15–25%)^4^. Histologically, endometritis has an acute inflammatory response that seriously damages uterine function. Recent research evidence suggests that excessive stimulation of endometrial epithelial cells may provoke adverse inflammatory responses in uterine tissue, which may cause endometrial damage and ultimately infertility^5^. Endometrial epithelial cells are important effector cells that the host defends against external stimuli, and they play a vital role in the pathogenesis of endometrial inflammation involved in endometrial injury^6^.

IFN-τ is a type I interferon secreted by ruminant trophoblast cells during embryo implantation^7^. In addition to its antiviral and antiproliferative effects, it has significantly low cytotoxicity and cross-species effects^8^. IFN-τ has an immunomodulatory function and has also been increasingly studied by researchers^9^. In recent years, studies have reported the biological characteristics of IFN-τ in terms of anti-inflammatory injury^10^. Among them, IFN-τ regulates the expression of Bo LA-I in the endometrium of dairy cows by activating STAT1 and STAT3 and downregulating TRAF3, thereby inducing the formation of the uterine immunosuppressive microenvironment^11^. Interestingly, previous research revealed that IFN-τ can inhibit the natural immune response in a mouse model of inflammatory injury^12^. Studies have confirmed that IFN-τ can play an important role in balancing the immune status of the endometrium by regulating the miRNA-mRNA interaction network^13^. Therefore, these studies prompted us to explore the potential regulatory effects of IFN-τ on endometrial inflammatory injury in dairy cows.

MicroRNAs are a class of evolutionarily conserved noncoding small molecule RNAs that guide the silencing complex (RISC) to degrade mRNA or hinder its translation by base complementation with the target gene mRNA^14,15^. MicroRNAs can regulate gene expression at the translation level. A large amount of evidence indicates that miRNAs are a new target for the treatment of various inflammatory diseases and are closely related to their pathogenesis^16^. The important role of miRNAs in the reproductive system of ruminants has been widely recognized in previous studies^17,18^. Recently miRNA expression profile analysis also revealed that uterine tissue miRNAs are involved in the occurrence and development of endometrial inflammatory injury in dairy cows^19^. However, it is not clear whether the mitigation effect of IFN-τ on the inflammatory injury of the endometrium can also be mediated by these differentially expressed miRNAs.

Studies have revealed that endometrial inflammatory damage is closely related to temporal and spatial changes in miRNAs in endometrial epithelial cells. Among them, miR-26a is highly conserved in mammals, and studies have shown that miR-26a is involved in the inflammatory response process. However, the mechanism by which miR-26a participates in the innate immune response during endometrial inflammatory injury is not clear. In the current study, we used endometrial epithelial cells to establish an inflammatory injury model in vitro and explored the biological function and regulatory mechanism of miR-26a in endometrial inflammatory injury. In addition, a mouse endometrial inflammatory injury model was established, further confirming the role of miR-26a in innate immune regulation.

## Methods and materials

### Primary reagents

Lipopolysaccharides (LPS) from *Escherichia coli *O55:B5 (Sigma USA) were used. IFN-τ (rOvIFN-τ, Lot: IFNT-29O, Creative Bioarry USA). Primary antibodies against AKT, PI3K and PTEN were obtained from Cell Signaling Technology (Bio-Techne Corporation Brands, USA). Cytokine (IL-1β, IL-6 and TNF-α) ELISA kits were obtained from Bioscience (Newark, DE, USA).

### Primary cell isolation and culture

A combination of mechanical methods and enzymatic digestion methods was used to culture bovine endometrial epithelial cells (bEECs or BEND). The anterior part of the uterine horn of 4 healthy pre-oestrus cows was collected 2–3 cm near the fallopian tube and placed in pre-cooled normal saline containing 2% (penicillin 100 IU/mL, streptomycin 1000 mg) bi-antibody. Under aseptic conditions, the separated uterine horns (1–2 cm) were placed in a beaker and washed repeatedly with DPBS solution without Ca^2+^ and Mg^2+^. The uterine horns were cut longitudinally in a sterile petri dish, rinsed repeatedly with DPBS containing 5% bi-antibody, and soaked in DPBS solution at 4 °C for 40 min. Then, the endometrial tissue was digested with 1% pronase at 4 °C overnight. The endometrial tissue was removed overnight, and the endometrial epithelial cells were scraped with a sterile scalpel. The cells were resuspended and washed with PBS 3 times, centrifuged at 1200 r/min for 5 min and this was repeated 3 times. The obtained cells were placed in DMEM/F12 medium containing 1% bi-antibody and 20% FBS, and the suspension cells were pipetted thoroughly. The cell count was adjusted to 1 × 10^5^ cells/mL. The cells were cultured in a 37 °C, 5% CO_2_ incubator for 24 h. After the cells were fully attached, the cell culture medium was changed once a day and cultured for 3 to 4 days to obtain primary cow endometrial epithelial cells.

### Identification of bovine endometrial epithelial cells

When the bEECs culture in the 6-well plate reached 60% ~ 70%, the cells were rinsed 3 times with PBS and fixed with 4% paraformaldehyde for 20 min. Then, the cells were incubated with 3% H_2_O_2_ for 15 min, washed 3 times with PBS for 3 min each time, blocked with 10% serum for 0.5 h, then the cells were incubated with a sufficient amount of primary antibody (CK-18, 1:1000) overnight at 4 °C and gently washed 3 times with PBS. The secondary antibody (1:750) was added dropwise, incubated at room temperature for 45 min, the cell nucleus was counter-stained with DAPI, to remove excess DAPI, the glass slide of the 6-well plate was taken out, filter paper was used to absorb the liquid on the cell slide, and the slide was mounted to prevent fluorescence quench. The cells were observed and images were collected under a fluorescence microscope.

### Experiment grouping and disposal

The bEECs cultured to the 4th generation were divided into the normal control group, LPS group, IFN-τ group and IFN-τ + LPS group; after the cells were synchronized, the cells were evenly seeded in a 6-well plate, and 4 mL culture medium was added to each well. The cells were placed in a cell incubator when the cell density reached 70–80%. IFN-τ at concentrations of 100 ng/mL was added for pretreatment for 1 h. The control group was treated with the same volume of PBS, and then the cells were stimulated with 2 μg/mL LPS for 24 h. After the stimulation time was over, cell samples were collected for subsequent experiments.

### Transfection of bEECs

After the bEECs were synchronized, the cells were evenly seeded in a 6-well plate and cultured in a cell incubator until the cell density reached 50 ~ 60%. The experiment was divided into miR-26a mimics, mimics NC, miR-26a inhibitors and inhibitors NC; PTEN specific siRNA (si-PTEN) or negative control siRNA (si-NC); all were designed and synthesized by Suzhou GenePharma Co., Ltd., and the transfection concentration was determined according to the recommended concentration in the reagent manual. The bEECs were transfected for 24 h and then stimulated with 2 μg/mL LPS for 6 h. After the stimulation time, the medium was changed, the culture was lasted for 24 h, and cell samples were collected for subsequent experiments. The transfection test procedure was performed according to the Invitrogen Lipofectamine 2000™ (Invitrogen, Carlsbad, California, USA) manufacturer's instructions.

### RT–qPCR assay

miR-26a mimic (50 nmoL) or inhibitor (100 nmoL) was transfected into bEECs. Then, 24 h after transfection the expression level of miR-26a was measured by qPCR (n = 6), and U6 snRNA was used as an internal control. The miRNA stem-loop primer and U6 were synthesized and purified by Nanjing GenScript Biotechnology Co., Ltd. The miRNA cDNA sequence was synthesized by reverse transcription using the miRNA reverse transcription kit of Suzhou Gema Gene Co., Ltd. After obtaining the cDNA of the reverse transcription product, the Hairpin-it™ microRNAs qPCR Quantitation Kit from Suzhou Gemma Gene Co., Ltd. was used for the qPCR assay; and the mRNA expression level of PTEN was detected by qPCR. The mRNA expression level of inflammatory factors was also detected and GAPDH was used as an internal reference.

### ELISA

ELISA kits were used to determine the levels of the inflammatory factors TNF-α, IL-1β and IL-6 in the supernatant of each group of cell cultures. The experimental steps were performed in accordance with the kit instructions.

### Immunofluorescence staining

In a 6-well culture plate, the slides that were mounted were immersed in PBS 3 times for 3 min each; the slides were fixed with 4% paraformaldehyde for 15 min, and the slides were immersed 3 times in PBS, 3 min each time; 0.5% Triton X-100 (prepared in PBS) was used to permeabilize the cells at room temperature for 20 min (this step was omitted if the antigen was expressed on the cell membrane); the slide was immersed 3 times in PBS, 3 min each time, absorbent paper was used to blot the PBS and placed on the slide. Normal goat serum was added dropwise and the slide was blocked at room temperature for 30 min; absorbent paper was used to absorb the blocking solution, a sufficient amount of diluted primary antibody was added to each slide and put it in a humid box, slides were incubated overnight at 4 °C; fluorescence was added in the dark. For application of the secondary antibody, PBST was used to soak the slides 3 times, 3 min each time, the excess liquid on the slides was absorbed with absorbent paper, and then the diluted fluorescent secondary antibody was added and the slides were incubated at 20–37℃ in a humid box for 1 h. The slides were then washed with PBST 3 times, 3 min each time; the nucleus was restained, DAPI was added dropwise and incubated for 5 min in the dark, the nucleus of the specimen was stained, the excess DAPI was washed off with PBST 5 min × 4 times; the liquid on the slide was blotted with absorbent paper, and an anti-fluorescence quencher was used to seal the slide. The slide was mounted with liquid, and then the slides were observed and images were collected under a fluorescence microscope.

### siRNA interference

The bEECs were grown to 1 × 10^5^ in a six-well plate and transfected with 100 nmoL siRNA duplex for 24 h using Lipofectamine 2000 (Invitrogen, USA) according to the manufacturer's instructions. si-PTEN or negative control (si-NC) was synthesized by Gene Pharma (Shanghai, China). After si-PTEN was transfected into bEECs for 24 h, they were then stimulated with 2 μg/mL LPS for 6 h. The medium was replaced with new medium and cultured for 24 h, and cell samples were collected. Immunofluorescence staining was performed to evaluate NF-κB p65 and PTEN, scale bar: 50 µm. DAPI's IOD was used as an internal reference, IPP 6.0 was used to calculate the IOD/area to indicate the level of p-p65.

### Western blotting

According to the manufacturer's instructions, the treated cells were lysed with RIPA buffer (Biosharp, China) and then centrifuged at 4 °C and 12,000×*g* for 15 min to extract total protein. The total protein concentration was determined using a BCA protein determination kit (Thermo Scientific, MA, USA). After adjusting the amount of each protein sample to 40 μg, the protein was separated using a 10% SDS-PAGE gel. The blot of the band of interest was cut prior to hybridization with the antibody. and then transferred to a PVDF membrane. The membrane was blocked with 5% skimmed milk for 2 h and incubated with the primary antibody (1:1000 dilution) at 4 °C overnight. The membrane was washed 3 times with TBST and incubated with the appropriate HRP-conjugated secondary antibody (1:3000 dilution) for 2 h at room temperature. A chemiluminescence kit was used to detect proteins, and Image-Pro Plus 6.0 gel analysis software was used to quantify the protein greyscale.

### Dual luciferase reporter gene experiment

HEK293T cells were grown and cultured before transfection, and 1 × 10^5^ cells/mL cell suspension was evenly inoculated in a 12-well plate. When the cell growth reached 50 ~ 60% and the cell growth status was well, Invitrogen's Lipofectamine™ 2000 reagents were used to co-transfect miR-26a mimic, mimic NC, and wild-type or mutant psiCHECK™-2-PTEN 3'-UTR recombinant plasmids. The wild-type or mutant PTEN 3'-UTR luciferase reporter vector and miR-26a mimic or mock NC were co-transfected into HEK293T cells. A dual luciferase reporter gene detection kit was purchased from Shanghai Yisheng Biotechnology Co., Ltd. The ratio of firefly luciferase/Renilla luciferase for each tube was calculated, and then the ratio of the control group was used as unit to obtain the relative luciferase activity of different treatment groups and calculate the ratio of Renilla luciferase activity to firefly luciferase activity. The group experiment was repeated 3 times.

### Animal experiments

Thirty healthy BALB/c mice aged 8 weeks were selected. They were randomly divided into 3 groups, each with 10 mice, namely, the control group, the LPS group and the IFN-τ + LPS group. The experimental animals were provided by the Experimental Animal Center of Huazhong Agricultural University. Among them, the breeding environment temperature was maintained at 24 ± 1 °C, the humidity was in the range of 45%-60%, and the mice were kept in a 12 h light/dark cycle every day. After feeding for one week, the mice in the model group were perfused with LPS at 2.5 mg/kg body weight in the uterus; the control group was given the same amount of phosphate buffered saline; in the IFN-τ + LPS group, 24 h after LPS perfusion, each animal IFN-τ (8 ng/g) was intraperitoneally administered, once every 8 h, for a total of 3 times. Eight hours after the last injection, the mice were anaesthetized with sodium pentobarbital, and animal tissue samples were collected and stored in a refrigerator at − 80 °C.

### Statistics and analyssis

The experimental results were statistically analyzed using the biostatistical software Graph Pad Prism 6.0, and the data were expressed as mean ± S.D. When comparing between two groups, independent sample Student's *t*-test was used to analyze the significance of differences; Dunnett's multiple comparison program or one-way analysis of variance (ANOVA) was used to perform multiple comparisons. *p < 0.05 indicates a significant difference, **p < 0.01 indicates a very significant difference, and all experiments are independently repeated 3 times.

### Ethical approval and consent to participate

This study was conducted in accordance with the guidelines of the Declara‑tion of Helsinki and was approved by the Animal Research Ethics Committee of Huazhong Agricultural University (HZAUMO-2015-12) were strictly implemented. All the authors consented to participate in this study.

### Consent for publication

Confirming the study is reported in accordance with ARRIVE guidelines (https://arriveguidelines.org).

## Results

### Cultivation and identification of bEECs

**Figure 1:**
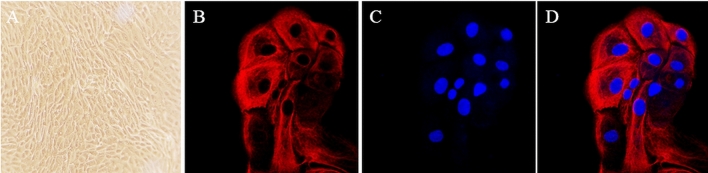
Culture and identification of bovine endometrial epithelial cells (bEECs). (**A**) Cell morphology under light microscope (× 100). (**B**) Expression of specific keratin 18 (red) in bEECs. (**C**) Blue is the nucleus stained by DAPI. (**D**) Combine (× 400).

The mechanical method combined with pronase digestion treatment successfully obtained primary dairy cow endometrial epithelial cells. Observed through a microscope, the cell morphology was similar to a paving stone-like distribution, the cytoplasm was transparent, the outline was clear and had a three-dimensional effect. The results are shown in Fig. 1A. Keratin 18 (CK-18) identification results showed that the number of positive cells reached more than 95%, and differential cell culture yielded higher purity bEECs. The results are shown in Fig. 1B–D.

### Establishment of inflammatory injury model

**Figure 2:**
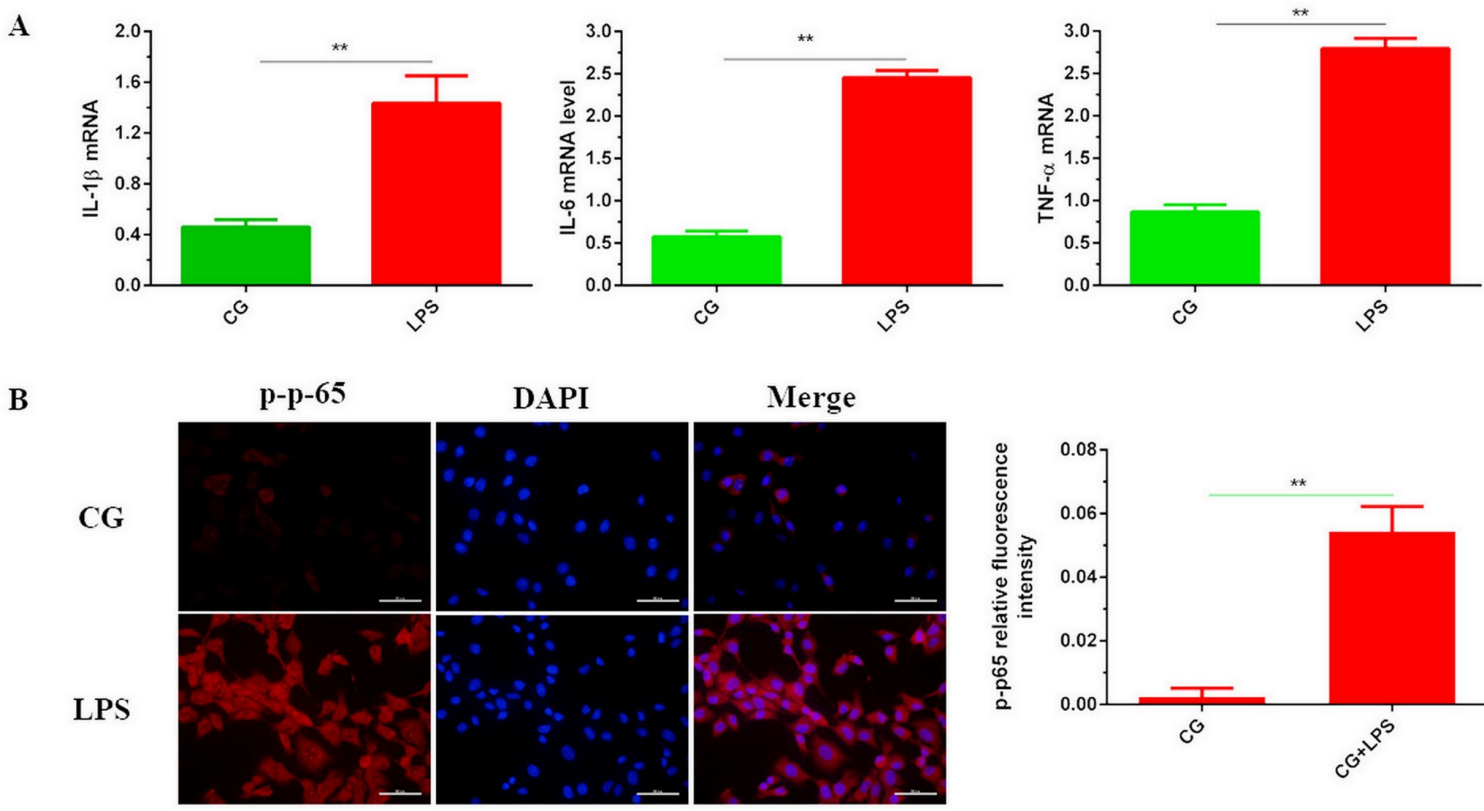
Construction of bEECs inflammatory injury model. (**A**) The levels of TNF-α, IL-1β and IL-6 were detected using qPCR in bEECs. CG is set as the control group; LPS is the model group. (**B**) The phosphorylation level of p65 was revealed by immunofluorescence experiment (× 400, scale = 50 μm); p-p65 was stained with red and the nucleus was blue. Image-Pro Plus (IPP) 6.0 software calculated IOD/area, and indicated the fluorescence intensity of p-p65. Data are expressed as mean ± S.D., three independent experiments. *p < 0.05; **p < 0.01.

The expression of TNF-α, IL-1β and IL-6 in bEECs was detected via qPCR. Compared with the control group, the mRNAs levels were significantly increased. The results are shown in Fig. 2A. In addition, the results of immunofluorescence experiments showed that p65 nuclear translocation significantly increased, and the phosphorylation level was significantly increased, confirming the credibility of the LPS-induced inflammatory injury model of bEECs. The results are shown in Fig. 2B. The results showed that the bEEC inflammatory injury model was successfully generated.

### miR-26a expression

**Figure 3:**
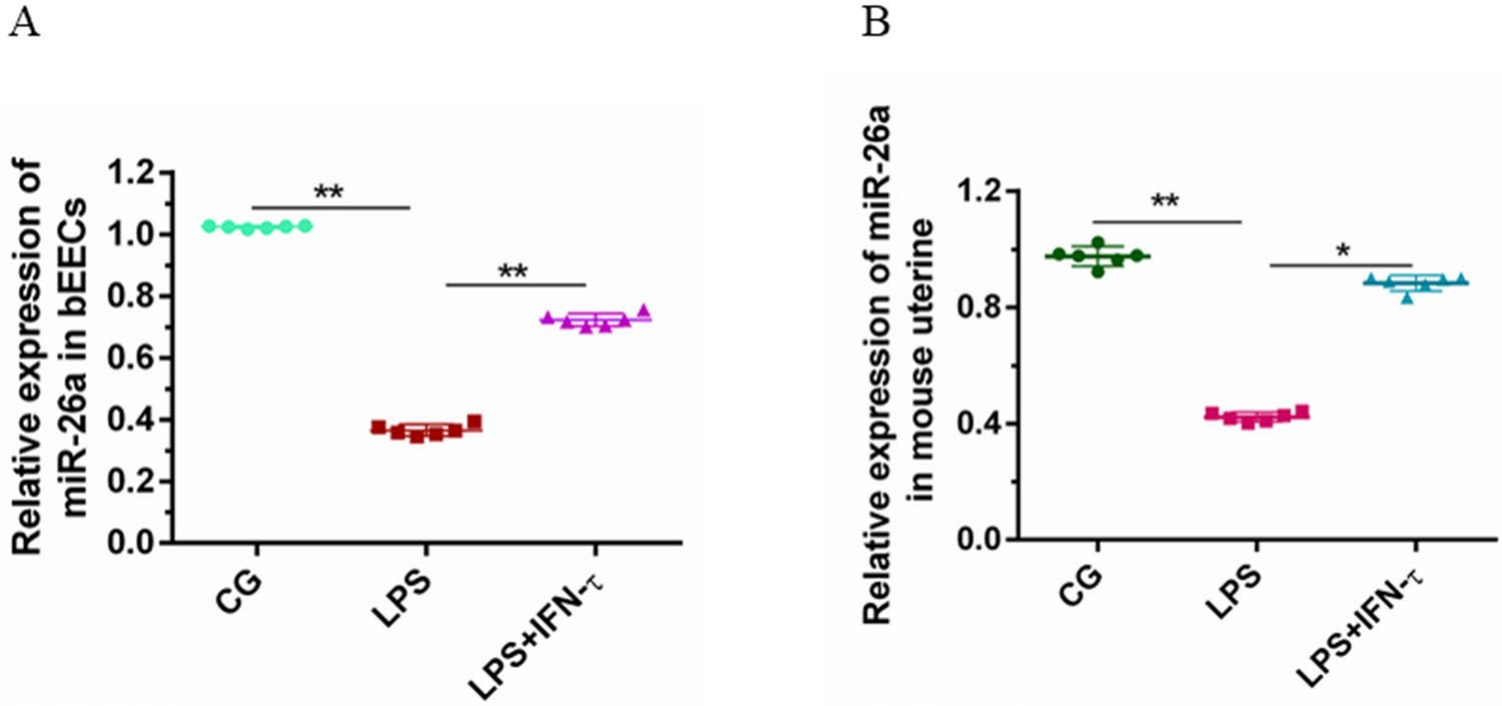
IFN-τ mediates miR-26a expression. (**A**) The expression of miR-26a in bEECs. (**B**) The total expression of miR-26a in mouse endometrial tissue. CG is the control group, LPS is the model group, and IFN-τ + LPS is the experimental group. Data are expressed as mean ± S.D., three independent experiments. *p < 0.05; **p < 0.01.

Small RNA qPCR results showed that the expression of miR-26a was significantly down-regulated in bEECs in the LPS group compared with the control group; however, the results in the IFN-τ + LPS group showed that IFN-τ could induce a significant up-regulation of miR-26a expression, which reversed the trend of expression in bEECs. The results are shown in Fig. 3A. The same conclusion was obtained by examining the expression of miR-26a in mouse uterine tissue (Fig. 3B).

### miR-26a inhibits cellular inflammatory response

**Figure 4:**
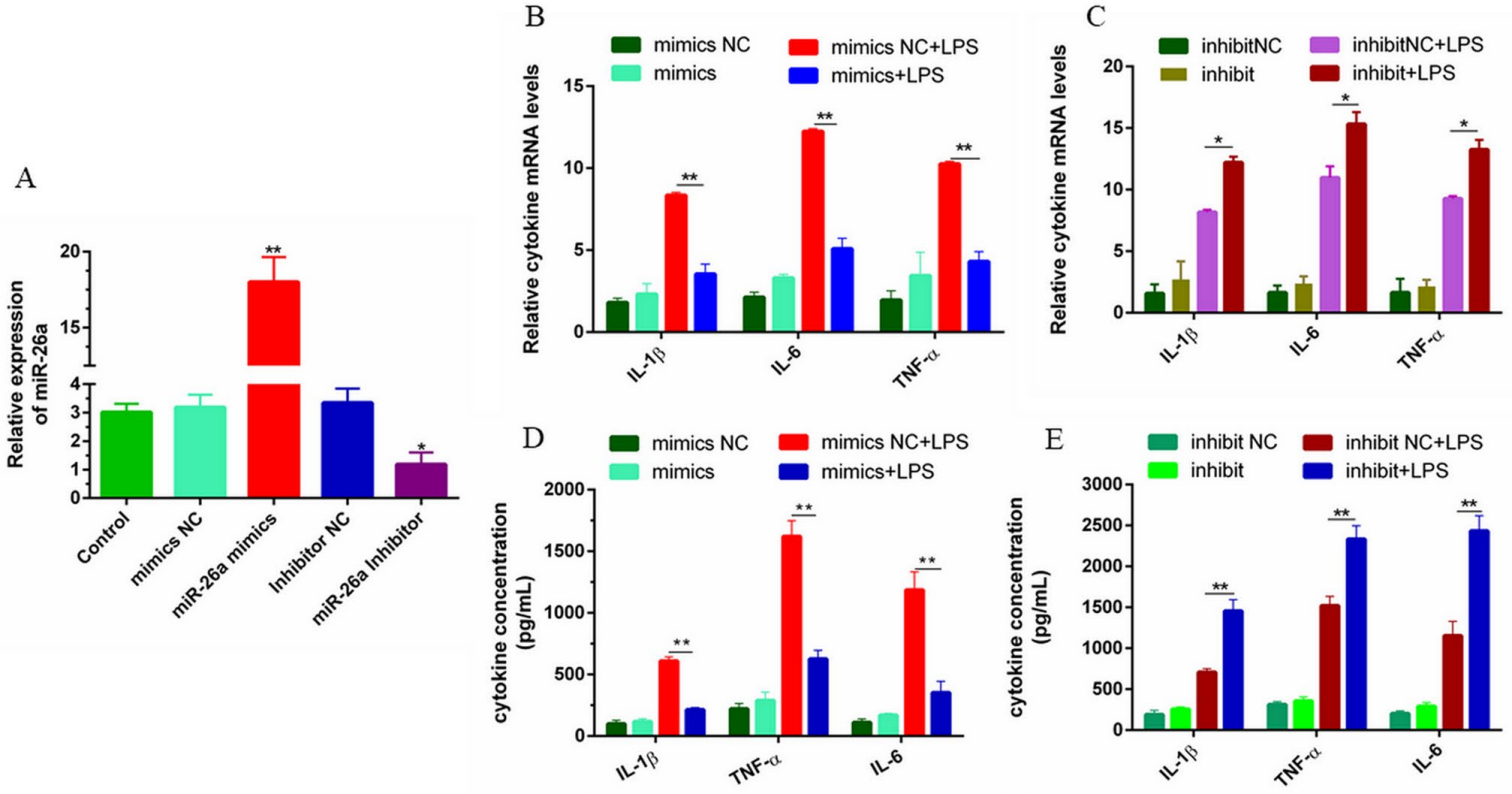
miR-26a suppresses LPS-mediated inflammatory cytokines in bEECs. (**A**) Liposomal transfection of miR-26a mimic (50 nM) or inhibitor (100 nM) into bEECs, 24 h after transfection, qPCR (n = 6) to detect the relative expression level of miR-26a using U6 as the internal control. (**B**, **C**) Cells were transfected with miR-26a mimics or inhibitors last for 24 h, and then stimulated with 2 μg/mL LPS for 24 h. The mRNA levels of inflammatory cytokines IL-1β, IL-6 and TNF-α were detected by qPCR using GAPDH as an internal reference. (**D**, **E**) ELISA detected the secretion levels of cytokines IL-1β, IL-6 and TNF-α. Data are expressed as mean ± S.D., three independent experiments. *p < 0.05; **p < 0.01.

**Figure 5:**
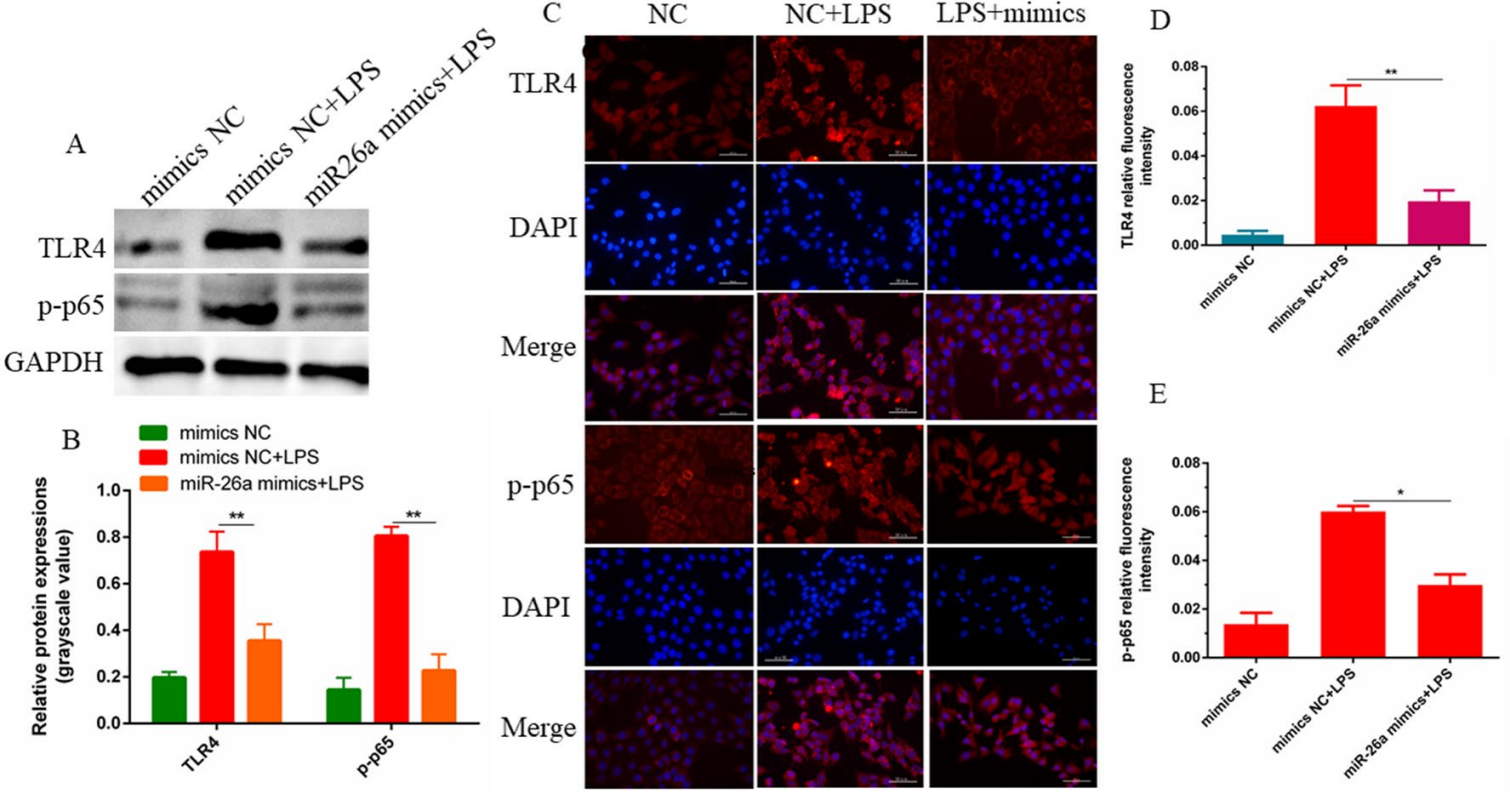
miR-26a negatively regulates the NF-κB pathway. (**A**, **B**) The miR-26a mimic and NC mimic were transfected into bEECs for 24 h. The phosphorylation level of NF-κB p65 and the expression level of TLR4 were determined by western blotting. Image-Pro Plus (IPP) 6.0 software calculated protein gray levels. GAPDH as a service reference. (**C**) Immunofluorescence (× 400) was used to assess the phosphorylation level of p65 and the expression of TLR4. (**D**, **E**) The IOD/area was evaluated by IPP 6.0 software, and revealed the p-p65 nuclear translocation and the fluorescence intensity of TLR4. Data are expressed as mean ± S.D., Three independent experiments. *p < 0.05; ** *p* < 0.01. the cropping of your gels and blots in the figure legends where relevant.

In this study, miR-26a mimics (mimics) were transfected into bEECs to overexpress miR-26a, and the expression of cytokines and proteins related to inflammation was detected. The results of qPCR and ELISA revealed that overexpression of miR-26a inhibited the secretion of the inflammatory factors IL-1β, IL-6 and TNF-α; in contrast, experimental results indicated that inhibiting the expression of miR-26a could promote the expression of related inflammatory factors (Fig. 4A–E). Western blot results showed that overexpression of miR-26a significantly down-regulated the protein levels of TLR4 and p-p65 induced by LPS (Fig. 5A,B). In addition, the results of immunofluorescence staining further confirmed the inhibitory effect of miR-26a on TLR4 and p-p65 (Fig. 5C–E). The above results indicate that miR-26a can negatively regulate the cellular inflammatory response induced by LPS by inhibiting the activation of TLR4 and downstream NF-κB. These results further indicated that miR-26a is involved in the inflammatory injury process caused by LPS and may be a key regulator of endometrial inflammatory injury.

### miR-26a sensitizes the PI3K/AKT pathway

**Figure 6:**
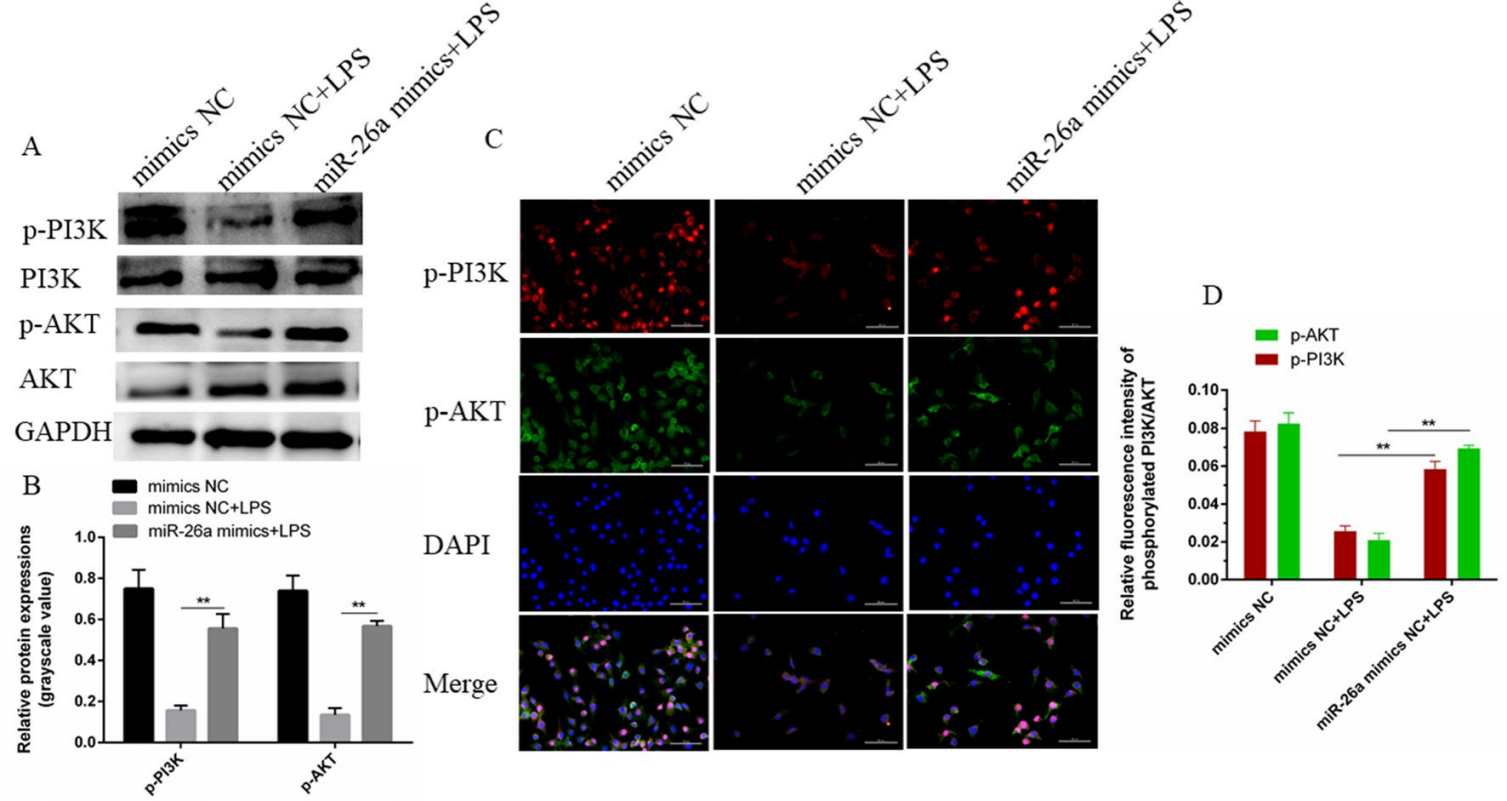
Overexpression of miR-26a activates PI3K/AKT signaling. (**A**) Western blot experiments indicated that the overexpression of miR-26a reversed the LPS-mediated expression down-regulation of p-PI3K and p-AKT. (**B**) The protein gray value was calculated by IPP6.0 software. GAPDH is set to the control internal reference. (**C**) Immunofluorescence suggested that (**A**) the same conclusion, and the overexpression of miR-26a can activate the PI3K/AKT axis (× 400, scale bar = 50 µm). (**D**) IPP6.0 calculated IOD/area to indicate the fluorescence intensity of PI3K and AKT phosphorylated proteins. p-PI3K shown in red fluorescence, p-AKT is green fluorescence, and the nucleus is blue; Merge (× 400, scale bar = 50 µm). Data are expressed as mean ± S.D., Three independent experiments. **p < 0.01. The cropping of your gels and blots in the figure legends where relevant.

To determine the effect of miR-26a on the PI3K/AKT pathway, we then analyzed the effect of miR-26a mimic transfection on PI3K/AKT pathway-related proteins. Western blot results showed that LPS stimulation significantly inhibited the protein levels of p-PI3K and p-AKT, but overexpression of miR-26a restored the LPS-induced inhibition of the levels of p-PI3K and p-AKT (Fig. 6A,B). In addition, the immunofluorescence staining results also showed that the overexpression of miR-26a blocked the down-regulation of p-PI3K and p-AKT induced by LPS (Fig. 6C,D). The above results confirm that miR-26a plays a positive regulatory role in the PI3K/AKT pathway.

### Verification of PTEN as a target of miR-26a

**Figure 7:**
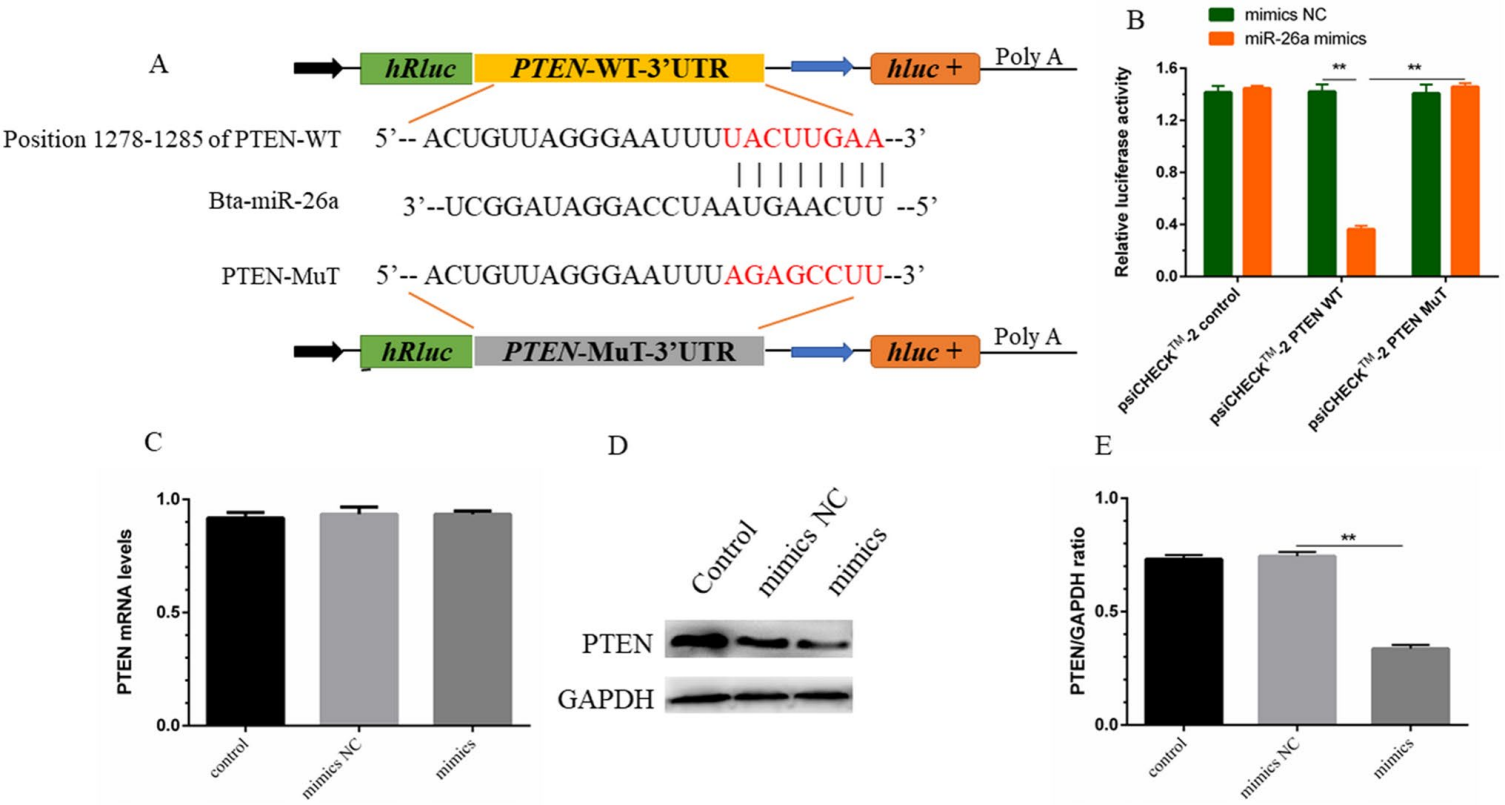
PTEN is the molecular target of miR-26a. (**A**) TargetScan 7.2 predicted the target sites of miR-26a and PTEN 3'UTR. (**B**) Co-transfect wild-type or mutant PTEN 3'-UTR luciferase reporter vector and miR-26a mimic or mimic NC into 293 T cells. The dual luciferase gene reporter system confirmed the ratio of Renilla activity/firefly activity, reflecting the luciferase activity. (**C**) miR-26a mimic, mimic NC or control were transfected into bEECs, after 24 h, the mRNA level of PTEN was measured by qPCR using GAPDH as an internal reference. (**D**) Cell processing is the same as (**C**). Western blot was used to detect the protein level of PTEN. GAPDH was used as an internal control. (**E**) IPP 6.0 software evaluated the gray value of PTEN. Three independent experiments. *p < 0.05; **p < 0.01. The cropping of your gels and blots in the figure legends where relevant.

miRNAs mainly participate in the intracellular signal cascade by inhibiting the translation of their target gene mRNA. This study further explored the target of miR-26a. PTEN was selected as a potential target of miR-26a through prediction analysis of biological databases (Fig. 7A). As an inhibitor of PI3K/AKT, PTEN not only plays a decisive role in tumour progression but also mediates inflammation and immune responses. First, a dual-luciferase reporter gene experiment was used to confirm whether PTEN is a molecular target of miR-26a. Experimental results showed that overexpression of miR-26a could significantly reduce the luciferase activity of the wild-type PTEN mRNA 3′-UTR vector without affecting the luciferase activity of the mutant vector (Fig. 7B); this result indicates that there is indeed a binding site for miR-26a in the 3′-UTR of PTEN mRNA. Further research found that miR-26a mimics significantly inhibited the expression of PTEN protein induced by LPS (Fig. 7D,E); however, the mRNA level of PTEN did not change significantly (Fig. 7C). In summary, these results confirmed that miR-26a targets and regulates the translation of PTEN and participates in the regulation of inflammatory damage in the endometrium of dairy cows.

### Knockdown of PTEN activates PI3K/AKT and inhibits NF-κB signaling

**Figure 8:**
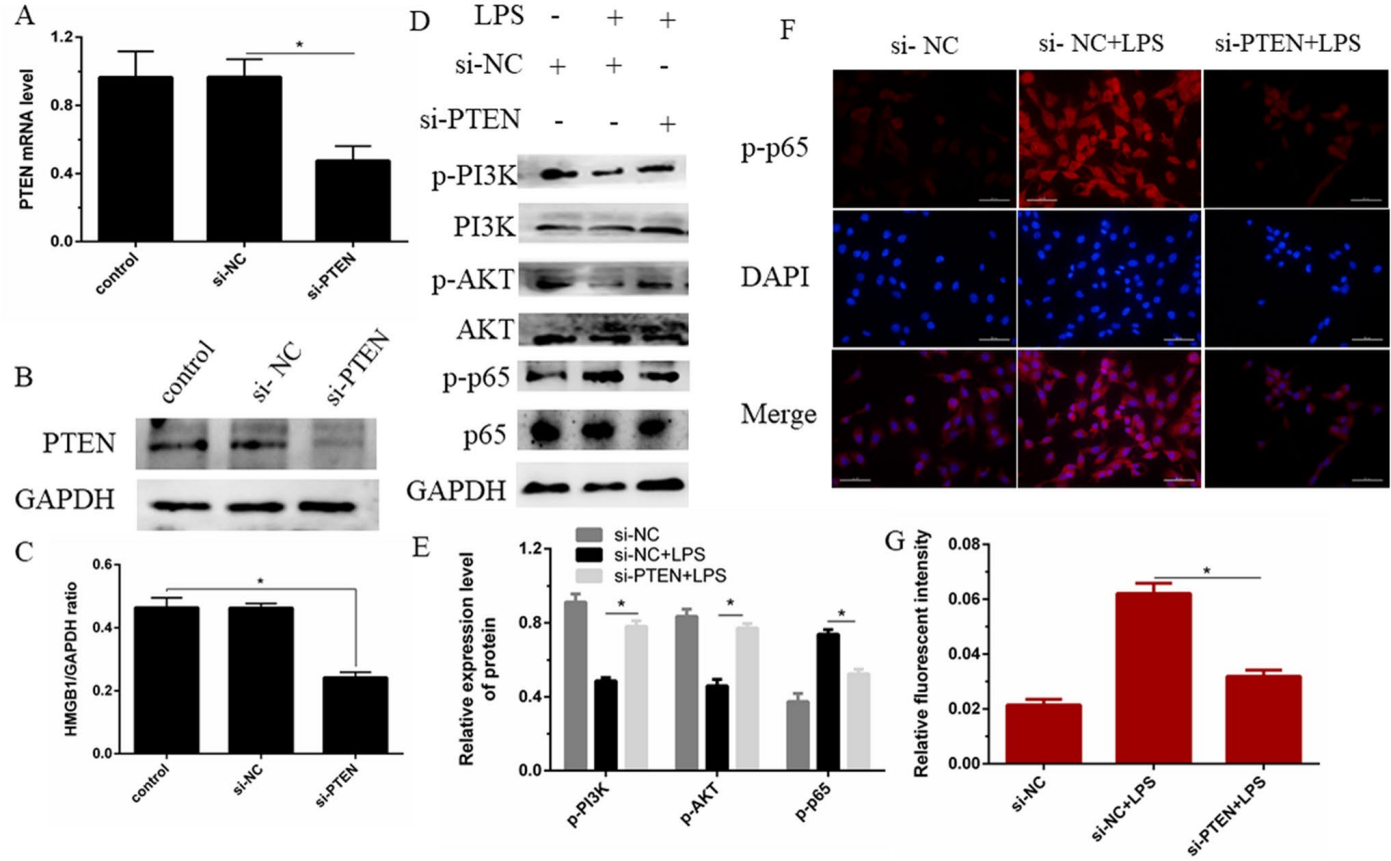
PTEN knockdown hinders the activation of NF-κB axis via activating PI3K/AKT signaling. (**A**) Transfected bEECs with PTEN-specific siRNA (si-PTEN) or negative control siRNA (si-NC) at a concentration of 200 nM after 24 h. qPCR to evaluate the mRNA level of PTEN. (**B**) The cell treatment was the same as (**A**), and the expression of PTEN was evaluated by western blotting. (**C**) IPP6.0 calculated the PTEN gray level, and GAPAH is the internal reference. (**D**, **E**) After the cells were transfected with si-PTEN or negative control siRNA (si-NC) for 24 h, the cells were treated with 2 µg/mL LPS for 24 h. Western blot was used to detect the phosphorylation levels of PI3K, AKT and p65. Calculated the protein gray level, GAPDH employs as an internal control (Supplementary information). (**F**, **G**) After the cells were processed in the same way, the nuclear translocation of p65 was detected by immunofluorescence. The area of IOD/area was calculated by IPP6.0 software. Data are expressed as mean ± S.D., Three independent experiments. *p < 0.05. The cropping of your gels and blots in the figure legends where relevant.

Studies have shown that knock down of PTEN by small interfering RNA (si-PTEN) promotes the activation of PI3K/AKT signaling, which in turn mediates the down-regulation of NF-κB signaling. Knock down of PTEN inhibited the phosphorylation of p65 induced by LPS, thereby blocking the pro-inflammatory effect of PTEN (Fig. 8A–E). Similarly, the immunofluorescence staining test further confirmed the knockdown of PTEN and inhibited the effect of p65 phosphorylation. (Fig. 8F,G). Based on the above experimental results, IFN-τ can target down-regulation of PTEN expression through miR-26a, activate the PI3K/AKT signaling pathway, and inhibit the activation of NF-κB.

### Protective effect of IFN-τ on inflammatory injury of endometrium in mice

**Figure 9:**
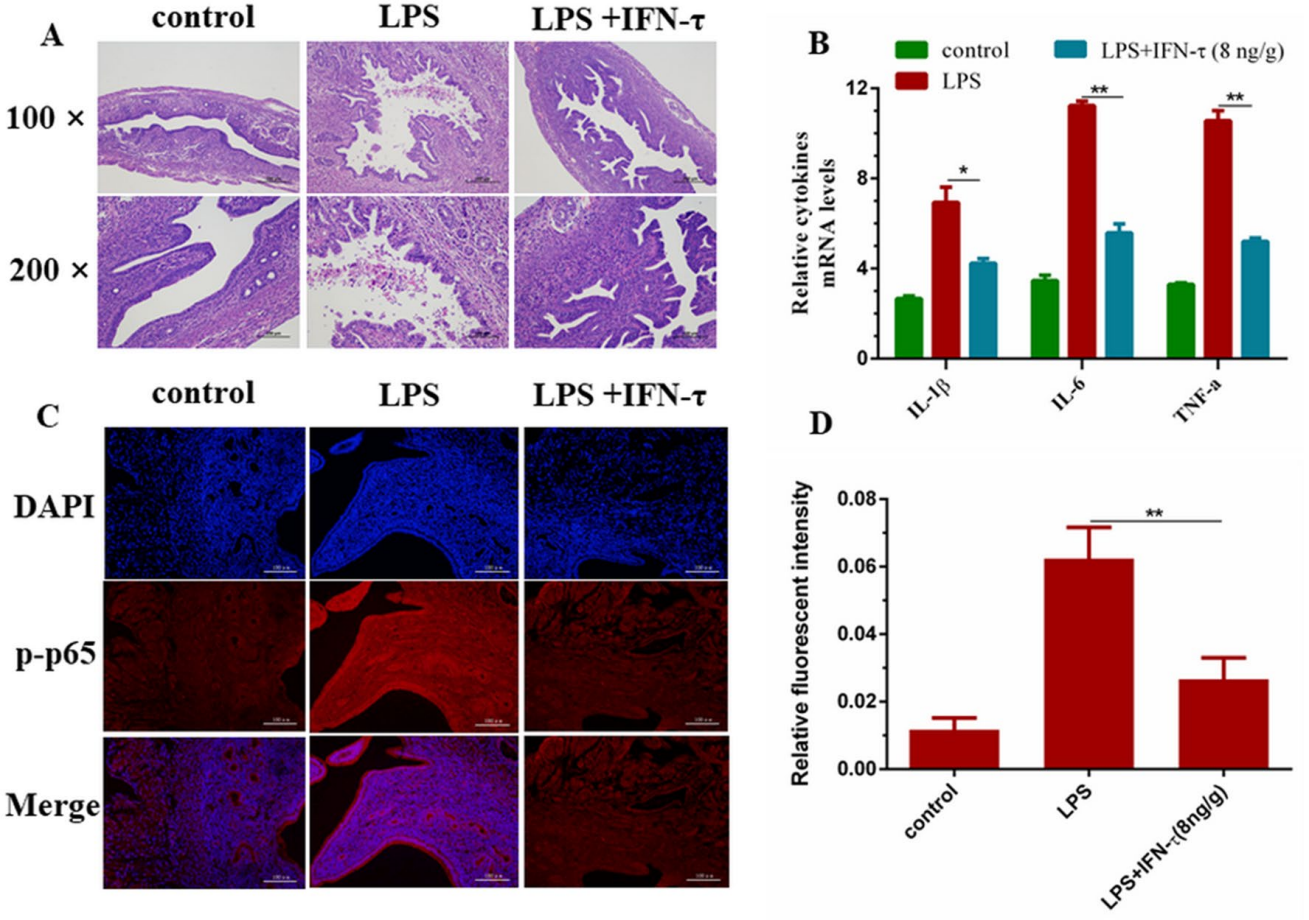
IFN-τ suppresses the inflammatory injury of the endometrium in mice. (**A**) The inflammatory injury of mice uterine samples were evaluated by H&E staining (n = 3). (**B**) qPCR evaluated the mRNA levels of related cytokines IL-1β, IL-6 and TNF-α in mice uterine tissue. (**C**, **D**) The results of immunofluorescence indicated that IFN-τ slowed down the phosphorylation of p65. Data are expressed as mean ± S.D., Three independent experiments. *p < 0.05; **p < 0.01.

A series of in vitro experiments have clearly confirmed that IFN-τ can activate the PI3K/AKT pathway by regulating miR-26a and reduce the inflammatory damage of the endometrium. In this study, a mouse endometrial inflammatory injury model was constructed by intrauterine infusion of LPS, and the effect of IFN-τ on endometrial injury in mice after intraperitoneal injection was explored. The results of hematoxylin and eosin (H&E) staining revealed that the IFN-τ group had significant changes in histomorphology compared with the LPS group, mainly manifested as severe inflammatory cell infiltration and tissue hemorrhage was inhibited (Fig. 9A). The qPCR results showed that, compared with the control group, the expression of related inflammatory factors was suppressed (Fig. 9B). The results of tissue immunofluorescence staining confirmed (Fig. 9C,D) that IFN-τ can inhibit the phosphorylation level of p65 in the inflammatory injury of mouse endometrium induced by LPS, and slow down the inflammatory injury of endometrial tissue.

## Discussion

Inflammatory damage to the endometrium of dairy cows is one of the main reasons leading to reproductive dysfunction, prolonged estrus, and infertility^20,21^. The proliferation of *Escherichia coli* in the uterus is the main cause of endometritis. During the dairy cow's production process, the environment in the uterus changes, and *E. coli* takes the opportunity to invade and colonize the uterus, causing intrauterine infection^22^. Antibiotics are often used in the initial treatment of endometritis, but the incidence is still as high as 20% to 50%, especially the emergence of drug-resistant strains, which has a huge negative impact^23,24^. Reports have shown that in the absence of immediate and effective treatment, the inflammatory injury of the endometrium will gradually evolve into a more serious disease^25,26^. Therefore, further revealing the mechanism of endometrial inflammatory injury, exploring new therapeutic targets, and providing new strategies for the treatment of endometrial inflammatory injury have become current research hotspots.

IFN-τ is specifically derived from ruminants and not only acts as a pregnancy signal for ruminants, but also regulates the body's inflammatory immune response by activating type I interferon pathways^11,27^. Research results indicate that IFN-τ not only has antiviral effects, but also has the effect of regulating inflammation^28^.The PTEN gene can regulate the synthesis of a special protein, and almost all tissues in the body contain this protein^28,29^. As a tumor suppressor, this protein regulates the cell division cycle by preventing cells from growing, dividing too fast, or dividing uncontrollably^28^. However, there is evidence so far that PTEN plays a key regulatory role in tissue inflammatory damage and repair^30^. This study disclosed that IFN-τ inhibited the expression of PTEN, thereby reversing the inflammatory effects of bEECs.

MicroRNA is a type of endogenous small RNA with a length of about 20–24 nucleotides, which has a variety of important regulatory effects in cells^31^. Research evidence supports that miR-26a is a key tumor factor inhibitor and participates in the regulation of the body's innate immune response^32,33^. Therefore, we believe that the anti-inflammatory effects of IFN-τ may regulate the expression and function of PTEN; and the expression of PTEN is very likely to be regulated by some specific miRNAs. In this study, we observed that IFN-τ mediated miR-26a expression is negatively correlated with PTEN in bEECs with inflammatory injury.

It has been demonstrated that PTEN negatively regulates PI3K/AKT signaling to regulate cell proliferation and apoptosis^34,35^. It has been reported that PTEN is up-regulated in inflammatory injury^36^. However, the role of PTEN in endometrial inflammatory injury needs to be further explored. In order to reveal the effect of miR-26a's post-transcriptional regulation of its target genes on the inflammatory response. The results of this study indicate that overexpression of miR-26a targeting PTEN 3'UTR negatively regulates the activity of PI3K/AKT signaling, thereby inhibiting the activation of NF-κB signaling. It leads to the down-regulation downregulation of the expression levels of related inflammatory factors IL-1β, IL-6 and TNF-α in the bEECs induced by LPS; at the same time, it inhibits the phosphorylation of related proteins. In this study, silencing the PTEN gene reversely verified that miR-26a targeted and regulated PTEN to activate the PI3K/AKT axis, thereby inhibiting the phosphorylation of NF-κB p65. We revealed that IFN-τ mediates the targeted regulation of miR-26a against LPS-induced inflammatory damage. It is well known that miRNAs regulate the expression of target genes in a diverse manner. Our study confirmed that the central role of IFN-τ in inhibiting LPS-induced inflammatory damage is through the posttranscriptional regulation of PTEN by miR-26a.

## Conclusion

This study concluded that IFN-τ can inhibit PTEN by promoting the expression of intracellular miR-26a, thereby inducing the activation of the PI3K/AKT signal axis and then negatively regulating the inflammatory response dominated by TLR4/NF-κB signaling.

## Supplementary Information

Supplementary Information.

## Data Availability

All data included in this study are available upon request by contact with the corresponding author.

## Abbreviations

bEECs: Bovine endometrial epithelial cells
LPS: Lipopolysaccharide
PTEN: Phosphatase and tensin homolog deleted on chromosome ten
PI3K/AKT: Phosphatidylinositide 3-kinases/protein kinase B
NF-κB: Nuclear factor kappa-B
DPBS: Dulbecco phosphate-buffered saline
DAPI: 4′,6-Diamidino-2-phenylindole
CK-18: Keratin 18
