# Supplementary material for: IFN-τ mediated miR-26a targeting PTEN to activate PI3K/AKT signalling to alleviate the inflammatory damage of bEECs
Source: Sci Rep. 2022 Jun 7;12:9410. doi: 10.1038/s41598-022-12681-9 (PMC9174273; doi:10.1038/s41598-022-12681-9)

**Gel scans from the main figures**

The original image of the repeated experiment.

**Fig. 5 A**


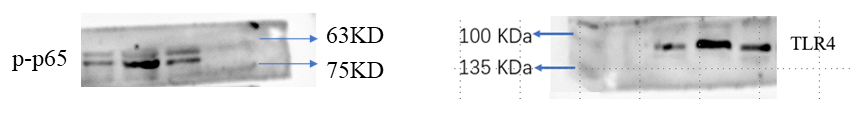


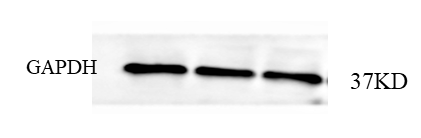


**Fig. 6 A**


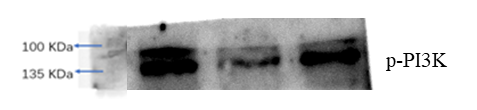


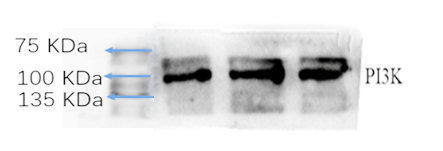


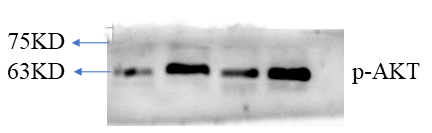


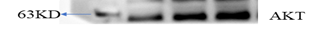


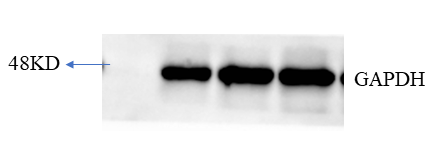


**Fig. 7 D**

Re-retain the sample and re-determine the experimental results of the target gene.

M: Marker 1: Control 2: mimics NC 3: mimics



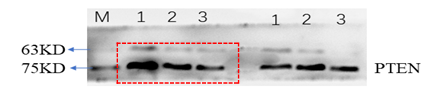



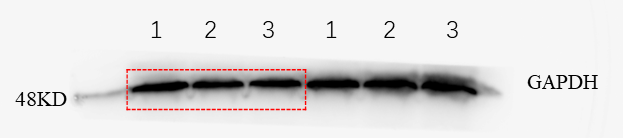


**Fig. 8 B**


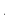

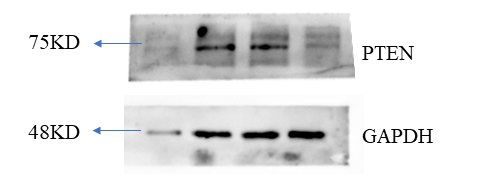


**Fig. 8 D**


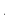
Used the original sample and re-determined the experimental results.

M: marker

1:si-NC 2: LPS 3: si-NC+LPS


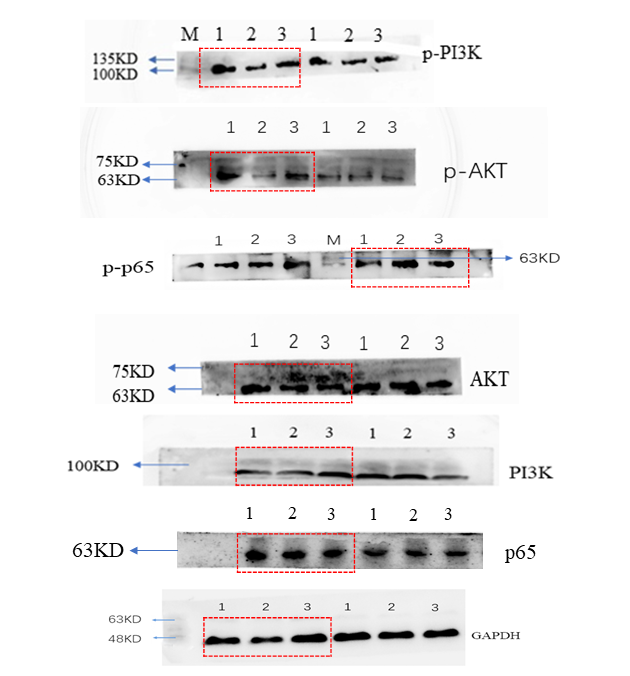

Supplement: Supplementary file 1 — Supplementary Information. [file 41598_2022_12681_MOESM1_ESM.docx]
